# Vitamin intake and pancreatic cancer risk reduction: A meta-analysis of observational studies

**DOI:** 10.1097/MD.0000000000010114

**Published:** 2018-03-30

**Authors:** Ying Liu, Xiaojie Wang, Xuejia Sun, Shengnan Lu, Shi Liu

**Affiliations:** a Department of Oncology, The 3rd Affiliated Hospital, Qiqihar Medical University, Qiqihar; b Heilongjiang Institute of Dermatology and Sexually Transmitted Disease, Harbin; c Department of Radiology, The 3rd Affiliated Hospital, Qiqihar Medical University; d Department of Ultrasound, The 2nd Affiliated Hospital, Qiqihar Medical University; e Department of General Surgery, The 3rd Affiliated Hospital, Qiqihar Medical University, Qiqihar, China.

**Keywords:** meta-analysis, pancreatic cancer, vitamin B12, vitamin D, vitamin intake

## Abstract

**Background:**

The relationship between vitamin intake and pancreatic cancer (PC) risk is disputed. We aimed to investigate the association between vitamin intake and the risk of PC via meta-analysis.

**Methods:**

We conducted a meta-analysis of studies concerning vitamin intake and the risk of PC from EMBASE, MEDLINE, and Cochrane Library. The search yielded 25 correlative studies including 1,214,995 individuals. The relative risks (RR) were examined by a random-effect model or fixed-effect model. Subgroup analysis, dose–response analysis, sensitivity analysis, meta-regression, and publication bias analysis were used to analyze studies.

**Results:**

The RR of PC in the highest vitamin intake group was 0.90 (95% confidence interval, 0.83–0.98) compared with that in the lowest vitamin intake in the prospective studies. Different increments of vitamin intake and the risk of PC were examined with dose–response analysis, and a decrease in the risk of PC was observed with vitamin D (25%) and vitamin B12 (27%).

**Conclusions:**

This meta-analysis found that vitamin intake can decrease the risk of PC, particularly vitamin D and vitamin B12.

## 1 Introduction

Pancreatic cancer (PC) is one of the most malignant cancers with a 5-year survival rate of about 5%.^[1]^ Almost 80% of PC patients are in the late stage at the first diagnosis in China,^[2]^ and incidence has been increasing in recent years.^[3]^ Therefore, efficacious preventive methods for PC, such as vitamin intake, have attracted worldwide attention. Vitamins have been suggested to prevent PC via several mechanisms.^[4]^ The preventive effects might be via up-regulation of p21 and p27 expression,^[5]^ increased activity of superoxide dismutase,^[6]^ cell cycle arrest at the G1 phase,^[7]^ suppression of NF-κB-mediated inflammatory pathways,^[8]^ down-regulation of Her2/ErbB2 expression,^[9]^ increased caspase-3 activity,^[10]^ or induction of Bax expression and activation EGR-1.^[11]^

Vitamin intake and the risk of PC have previously been reported. However, retrospective case–control studies, cohort studies, randomized placebo-controlled trials (RCTs), and some meta-analyses^[12–22]^ have had various results for the relationship between vitamin intake and the risk of PC. Therefore, we aimed to investigate the association between vitamin intake and the risk of PC via meta-analysis.

## 2 Methods

### 2.1 Search strategy

Studies investigating vitamin intake and PC were searched in EMBASE, MEDLINE, and Cochrane Library through March 30, 2015. Search terms were (pancreas OR pancreatic) AND (cancer OR carcinoma OR neoplasm) AND (vitamin OR food OR diet OR nutrition). References of the retrieved papers were hand-searched for potentially correlative papers. Two authors searched the studies and retrieved papers independently. Disagreements were solved by deliberation with other authors. This study was approved by the Ethics Committee of Qiqihar Medical University.

### 2.2 Study selection

The inclusion criteria of retrieved papers were case–control, placebo–control, or cohort design; vitamin intake as the independent variable of interest; PC as the dependent variable of interest plus reported PC incidence; and reported odds ratio (OR), relative risk (RR), or hazard ratio with the corresponding 95% confidence interval (CI). Nonhuman studies, mechanistic research, and review articles were excluded.

### 2.3 Data extraction

Two authors read the retrieved papers and extracted data independently from the studies according to the selection criteria. Disagreements were solved by deliberation with other authors. The following information was extracted from each paper: first author's last name, year of publication, study design, geographic location, the age and sex of participants, follow-up period, the size of study, type and doses of vitamins, RR or OR with 95% CI for vitamin intake, and PC risk. When 2 or more papers concerned the same study, the paper with the most data was used in this study.

### 2.4 Quality assessment

Two authors independently evaluated the quality of retrieved studies according to the Newcastle–Ottawa scale. The retrieved papers were evaluated based on selection of cohorts (0–4 points), comparability of cohorts (0–2 points), and exposure/outcome of the participant (0–3 points). Studies with 7 to 9 points were marked as “high quality.”

### 2.5 Statistical analysis

RRs or ORs with 95% CI and their standard errors were obtained from the studies to assess the relationship between vitamin intake and the risk of PC. The random-effect model was used to combine RRs or ORs with 95% CI concerning both intra- and inter-study variation (τ^2^). I^2^ was used to evaluate heterogeneity among studies including here, and I^2^ values of 25%, 50%, and 75% were considered low, moderate, and high heterogeneity, respectively. A fixed-effect model was utilized if I^2^ values <50%, otherwise a random-effect model was selected. Meta-regression of the variables of study design, vitamin dose, and geographic area of study was employed to assess heterogeneity among all included studies. The influence of grouping on total results was evaluated by subgroup stratification analysis. Potential causes of heterogeneity were estimated by the sensitivity analysis. Publication bias was evaluated by means of funnel plots and Egger test. This meta-analysis was carried out with Rev Man 5.3 or Stata 12.1, and *P* < .05 was considered statistically significant.

## 3 Results

### 3.1 Search results and study characteristics

**Figure 1:**
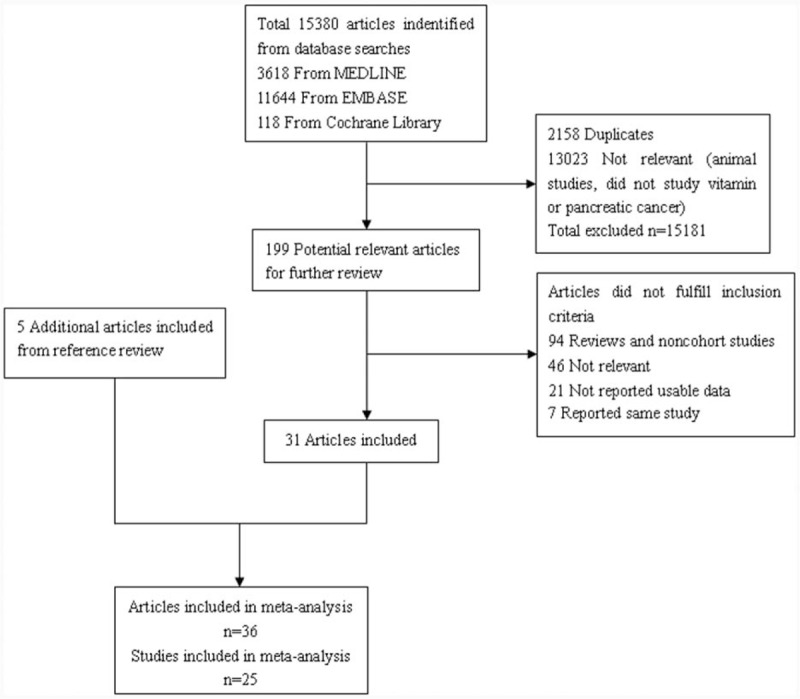
Flow diagram of study selection.

In this meta-analysis, we retrieved 25 studies including 1,213,821 participants published from 1991 to 2014 (Fig. 1). In the identified studies (Table 1), 10 were population-based case–control studies,^[23–34]^ 4 were hospital-based case–control studies,^[35–39]^ 2 were RCTs,^[40–44]^ 9 were cohort studies,^[45–56]^ 11 were prospective studies,^[40–56]^ and 14 were retrospective studies.^[23–39]^ The number of participants ranged from 305^[34]^ to 537,218^[45]^ and PC cases ranged from 79^[44]^ to 2383.^[45]^ Quality scores of included case–control and cohort studies ranged from 7 to 9 with an average score of about 8. The quality of RCTs was also estimated (data not shown).

**Table 1:**
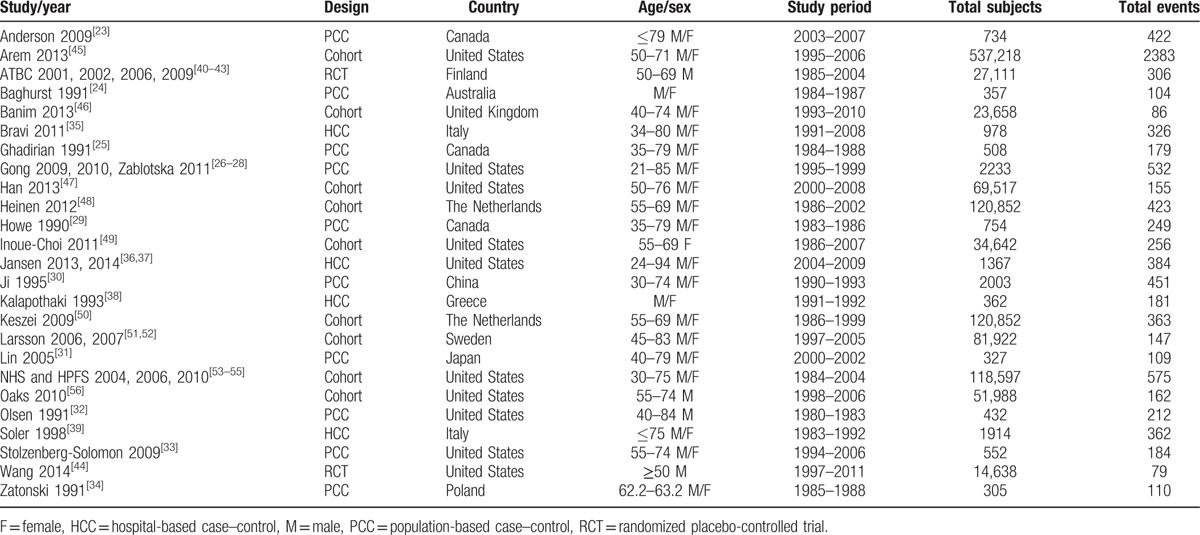
Characteristics of the included studies.

### 3.2 Vitamin intake and pancreatic cancer risk

**Figure 2:**
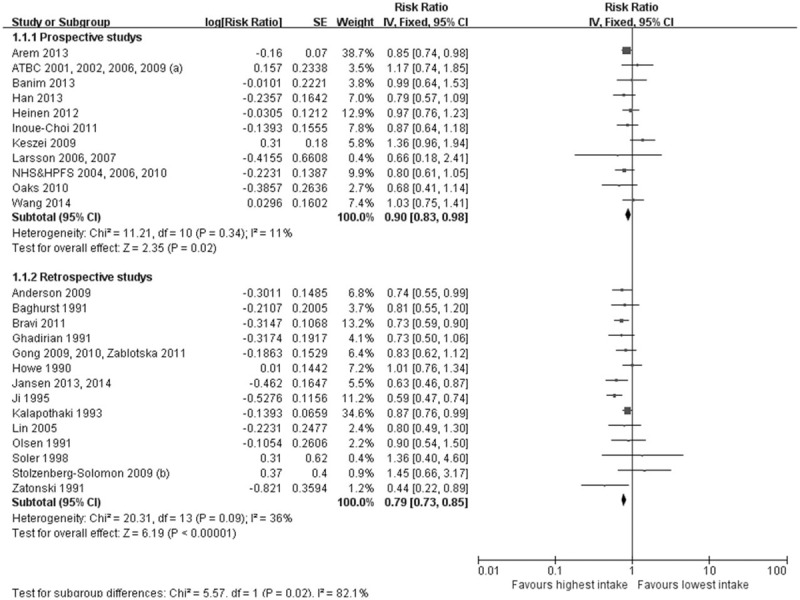
Forest plot of vitamin intake and risk of pancreatic cancer. Squares or diamonds to the left of the solid vertical line indicate benefit with vitamin intake.

A fixed-effect model was used and the combined multivariable-adjusted RR were 0.90 (95% CI: 0.83–0.98) and 0.79 (95% CI: 0.73–0.85) for the highest vitamin intake group compared with the lowest intake group in the prospective studies and retrospective ones, respectively (Fig. 2). Among the 25 studies, an opposite association between vitamin intake and PC risk was observed in 19 studies^[23–28,30–32,34–38,45–49,51–56]^ and was statistically significant in 7 studies.^[23,30,34–38,45]^ No significant heterogeneity was observed among included studies (*P* < .00001, I^2^ = 36% among retrospective studies; *P* < .05, I^2^ = 11% among prospective studies). These results demonstrate that moderate vitamin consumption can reduce the risk of PC.

### 3.3 Dose–response meta-analysis

**Figure 3:**
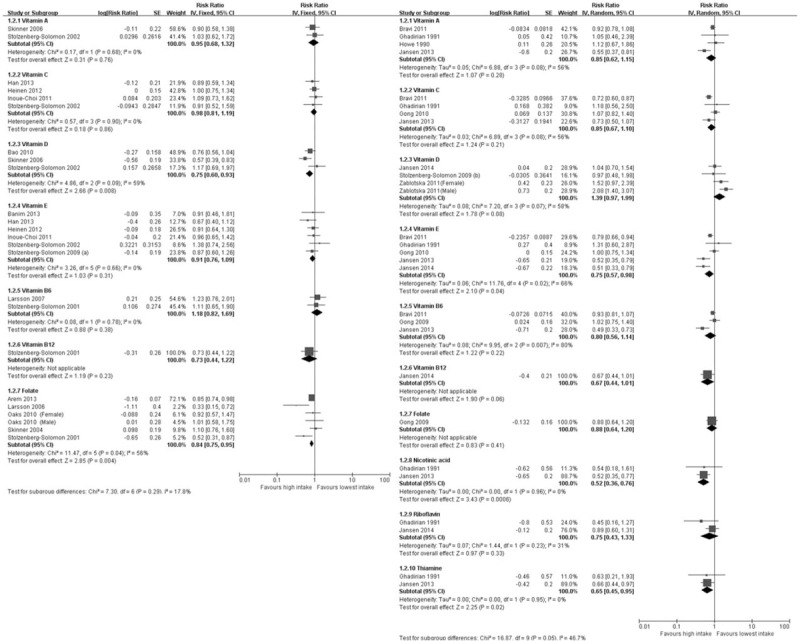
Forest plot of dose–response meta-analysis. Squares or diamonds to the left of the solid vertical line indicate benefit with vitamin intake. Left forest plot indicates prospective studies and the right one indicates retrospective studies.

In prospective studies, the multivariable-adjusted RR of vitamin D (10 μg/d) intake was 0.75 (95% CI: 0.60–0.93) with moderate heterogeneity (*P* = .008, I^2^ = 59%) in the 3 included studies. The multivariable-adjusted RR of vitamin B12 (10 μg/d) intake was 0.73 (95% CI: 0.44–1.22) in 1 included study. Figure 3 details the dose–response meta-analysis data.

In retrospective study, the multivariable-adjusted RR of vitamin E (10 mg/d) intake was 0.75 (95% CI: 0.57–0.98) with moderate heterogeneity (*P* = .04, I^2^ = 66%) in the 5 included studies. The multivariable-adjusted RR of vitamin B12 (10 μg/d) intake was 0.67 (95% CI: 0.44–1.01) in 1 included study. The multivariable-adjusted RR of nicotinic acid (30 mg/d) intake was 0.52 (95% CI: 0.36–0.76) without heterogeneity (*P* = .0006, I^2^ = 0%) in the 2 included studies. The multivariable-adjusted RR of riboflavin (3 mg/d) intake was 0.75 (95% CI: 0.43–1.33) without significant heterogeneity (*P* = .33, I^2^ = 31%) in the 2 included studies. The multivariable-adjusted RR of the thiamine (2 mg/d) intake was 0.65 (95% CI: 0.45–0.95) without heterogeneity (*P* = .02, I^2^ = 0%) in the 2 included studies. Figure 3 details the dose–response meta-analysis data.

### 3.4 Subgroup analysis

#### 3.4.1 Study design

The multivariable-adjusted RR of the prospective studies was 0.90 (95% CI: 0.83–0.98), which demonstrated that vitamin intake can moderately reduce the risk of PC. The multivariable-adjusted RR of the retrospective studies was 0.79 (95% CI: 0.73–0.85), which suggested that vitamin intake can significantly reduce the risk of PC. Details of the subgroup analysis are shown in Table 2.

**Table 2:**
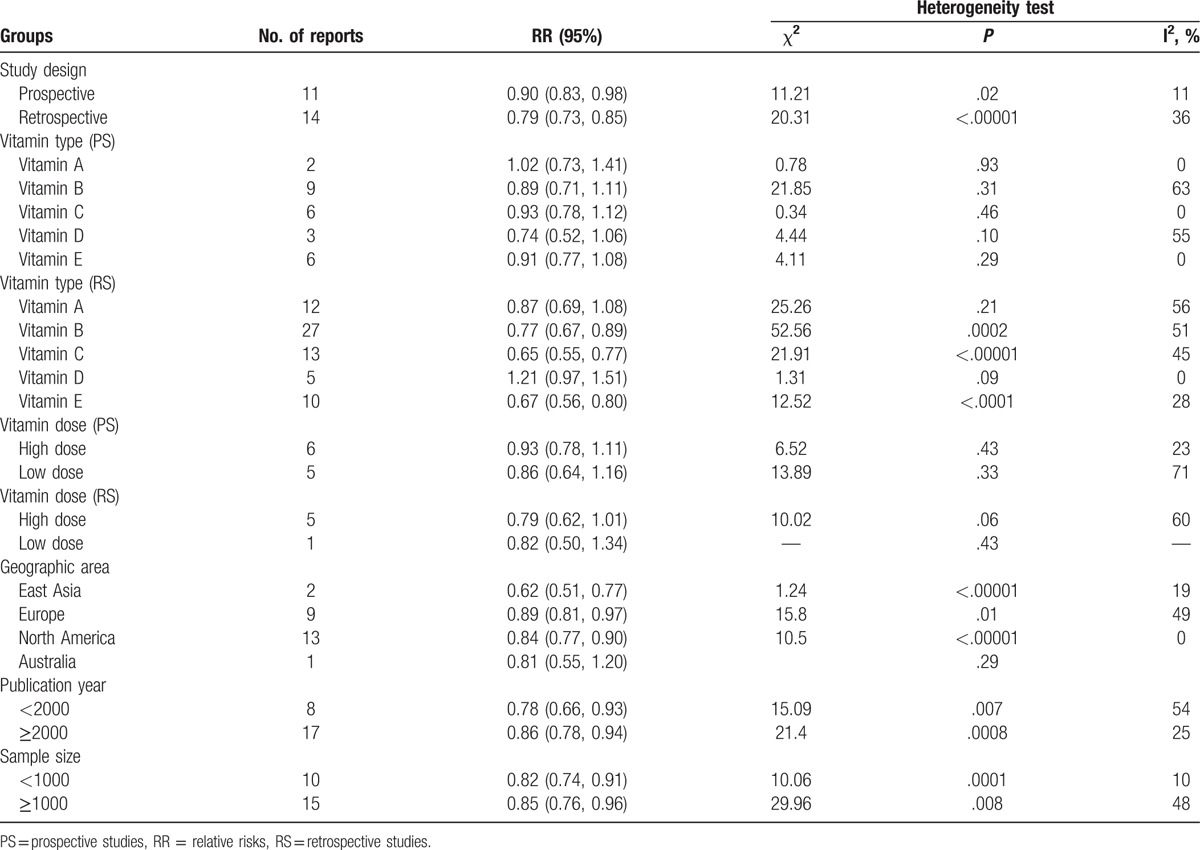
Subgroup analyses of vitamin intake and pancreatic cancer risk.

#### 3.4.2 Geographic area

The combined RR was 0.84 (95% CI: 0.77–0.90) for research carried out in North America,^[23,25–29,32,33,36,37,44,45,47,49,53–56]^ 0.89 (95% CI: 0.81–0.97) for research carried out in Europe,^[34,35,38–43,46,48,50–52]^ 0.62 (95% CI: 0.51–0.77) for research carried out in East Asia,^[30,31]^ and 0.81 (95% CI: 0.55–1.20) for research carried out in Australia.^[24]^ These results demonstrated that vitamin intake can moderately decrease the risk of PC (Table 2).

#### 3.4.3 Vitamin dose

**Figure 4:**
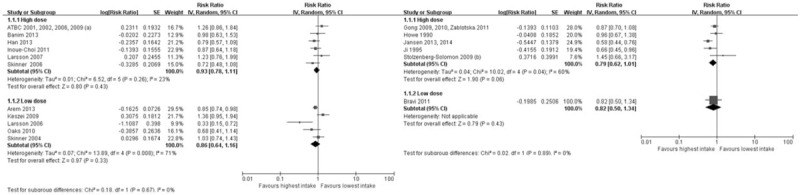
Forest plot of high-dose versus low-dose vitamin intake and risk of pancreatic cancer. Squares or diamonds to the left of the solid vertical line indicate benefit with vitamin intake. Left forest plot indicates prospective studies and the right one indicates retrospective studies.

In prospective studies, there was no significant difference in PC risk in the high-dose group compared with the low-dose (Fig. 4; Table 2). The combined RR was 0.93 (95% CI: 0.78–1.11) in participants who were given 2 or more times the vitamin dosage than the standard vitamin intake level in 6 studies (high-dose group). The combined RR was 0.86 (95% CI: 0.64–1.16) in participants who were given doses under the standard vitamin intake level in 5 studies (low-dose group).

In retrospective studies, there was no significant difference in PC risk in the high-dose group compared with the low-dose (Fig. 4; Table 2). The combined RR was 0.79 (95% CI: 0.62–1.01) in participants who were given 2 or more times the vitamin dosage than the standard vitamin intake level in 5 studies (high-dose group). The RR was 0.82 (95% CI: 0.50–1.34) in participants who were given doses under the standard vitamin intake level in 1 study (low-dose group).

#### 3.4.4 Vitamin type

In prospective studies, the combined RR of vitamin A or retinol intake and PC risk was 1.02 (95% CI: 0.73–1.41).^[41,54]^ The combined RR of B family vitamin intake and PC risk was 0.89 (95% CI: 0.71–1.11).^[40,45,50–53,56]^ The combined RR of vitamin C intake and PC risk was 0.93 (95% CI: 0.78–1.12).^[41,44,46–49]^ The combined RR of vitamin D intake and PC risk was 0.74 (95% CI: 0.52–1.06).^[42,54,55]^ The combined RR of vitamin E intake and PC risk was 0.91 (95% CI: 0.77–1.08).^[43,44,46–49]^ These findings are summarized in Table 2.

In retrospective studies, the combined RR of vitamin A or retinol intake and PC risk was 0.87 (95% CI: 0.69–1.08).^[24,25,28–32,34–36,38,39]^ The combined RR of B family vitamin intake and PC risk was 0.77 (95% CI: 0.67–0.89).^[23–26,32,35–38]^ The combined RR of vitamin C intake and PC risk was 0.65 (95% CI: 0.55–0.77).^[23–25,27,29–32,34–36,38]^ The combined RR of vitamin D intake and PC risk was 1.21 (95% CI: 0.97–1.51).^[24,28,33,35,37]^ The combined RR of vitamin E intake and PC risk was 0.67 (95% CI: 0.56–0.80).^[24,25,27,29–32,35–37]^ These findings are summarized in Table 2.

### 3.5 Sensitivity analyses and meta-regression

In prospective study group, the combined RR was 0.91 (95% CI: 0.82–1.00) after 3 studies^[44,49,55]^ were excluded owing to not adjusting for dietary factors or total energy intake with a moderate level of heterogeneity (*P* = .06, I^2^ = 28%). The combined RR was 0.89 (95% CI: 0.82–0.98) among 10 studies adjusted for smoking with a moderate level of heterogeneity (*P* = .01, I^2^ = 14%).^[40–43,45–56]^ The combined RRs were 0.88 (95% CI: 0.81–0.96) to 0.94 (95% CI: 0.84–1.04) after any single study was excluded, which did not affect the final result.

In retrospective study group, the combined RR was 0.79 (95% CI: 0.73–0.85) after 1 study^[39]^ was excluded owing to not adjusting for dietary factors or total energy intake with a moderate level of heterogeneity (*P* < .00001, I^2^ = 39%). The combined RR was 0.78 (95% CI: 0.72–0.84) among 13 studies adjusted for smoking with a moderate level of heterogeneity (*P* < .00001, I^2^ = 33%).^[23–32,34–39]^ The combined RRs were 0.75 (95% CI: 0.68–0.82) to 0.82 (95% CI: 0.75–0.88) after any single study was excluded, which did not affect the final result.

Meta-regression analysis demonstrated that study design (*P* = .005) included significant sources of heterogeneity. Study design alone explained 44.52% of the τ^2^ in the meta-regression analyses.

### 3.6 Publication bias

**Figure 5:**
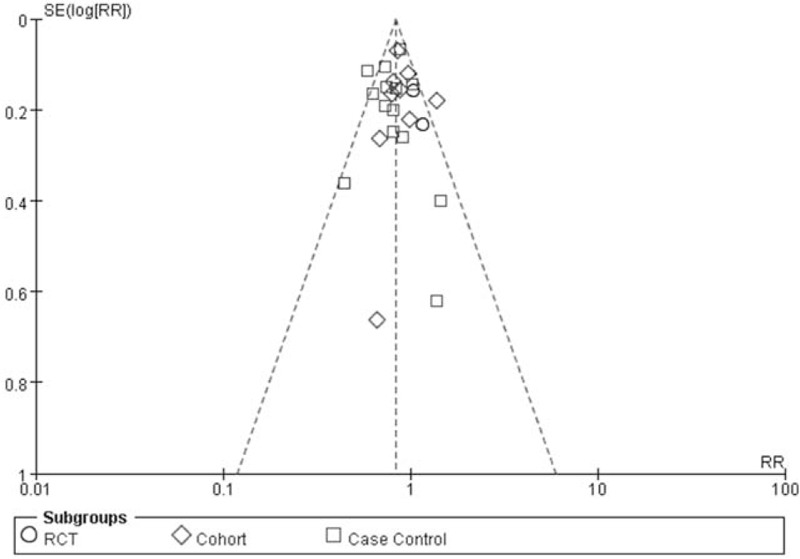
Funnel plot of relative risk of studies.

No unambiguous asymmetry was detected in the funnel plot (Fig. 5) and no publication bias was observed in the Egger test (*P* = .764).

## 4 Discussion

This meta-analysis included more than 1.2 million human participants and 8000 PC cases. We found that vitamin consumption can moderately decrease the risk of PC. Daily consumption of 10 μg/d of vitamin B12 or vitamin D can dramatically reduce the incidence of PC, 27% for vitamin B12 and 25% for vitamin D in the dose–response meta-analysis.

Several RCTs and observational studies have explored the association of vitamin consumption and the risk of PC. Some studies reported that vitamin consumption may be correlated with PC incidence.^[26,27,30–32,34–37,40,43,51,54,56]^ However, others found that vitamin consumption had no influence on the incidence of PC.^[38,44,48,50,53]^ Vitamin intake may also have a negative effect on the prevention of PC.^[28,39,42]^ The discrepancy of study design, type and dosage of vitamin intake, method used to estimate vitamin intake, and the time of follow-up may contribute to the different results among the studies.

Some meta-analyses have reported a preventive effect of vitamins on PC.^[12–22]^ The results of some of them suggested that vitamin intake can reduce the risk of PC,^[13,17,19–21]^ which echo this study. Nevertheless, several other studies reported that vitamins cannot decrease the risk of PC and may increase the risk.^[12,14–16,18,22]^ Differences in vitamin dosages used in the latter studies, inclusion of retrospective case–control studies, and inclusion of high-risk individuals, such as long-time chronic smokers, may contribute to discrepancies between their conclusions and ours.

Many studies^[23–26,28,32,33,35–38,40,42,45,50–56]^ have found that nonantioxidant vitamins may help prevent PC. However, some studies^[23–25,27–32,34–39,41,43,44,46–49,54]^ have suggested that antioxidant vitamins, such as vitamins A, C, and E, may influence the prevention of PC. Nevertheless, it is difficult to separate antioxidant vitamins from nonantioxidant vitamins in the daily diet. Therefore, we combined antioxidant and nonantioxidant vitamins together in this meta-analysis.

This meta-analysis demonstrated that vitamins can moderately reduce the incidence of PC. We found that the RR was 0.79 (95% CI: 0.73–0.85) in retrospective studies; however, it was 0.90 (95% CI: 0.83–0.98) in the prospective ones. It suggested that vitamin consumption can moderately decrease the risk of PC. In retrospective study, the RR of vitamin E intake was 0.75 (95% CI: 0.57–0.98), the RR of vitamin B12 intake was 0.67 (95% CI: 0.44–1.01), the RR of nicotinic acid intake was 0.52 (95% CI: 0.36–0.76), the RR of riboflavin intake was 0.75 (95% CI: 0.43–1.33), and the RR of the thiamine intake was 0.65 (95% CI: 0.45–0.95). Nevertheless, the prospective studies suggested that the consumption of vitamin D (10 μg/d; RR: 0.75; 95% CI: 0.60–0.93) and vitamin B12 (10 μg/d; RR: 0.73; 95% CI: 0.44–1.22) can decrease the risk of PC. These dose–response meta-analysis data recommended that daily consumption of 10 μg/d of vitamin B12 or vitamin D can dramatically reduce the incidence of PC, 27% for vitamin B12 and 25% for vitamin D. Some in vitro studies have suggested that nicotinic acid, thiamine, and vitamin B12 can prevent PC. Pour and Lawson^[57]^ suggested that nicotinic acid can inhibit pancreatic carcinogenesis in a hamster model. Zhang et al^[58]^ reported that nicotinamide prohibits proliferation and enhances chemosensitivity in PC cells; and Hanberry et al^[10]^ reported that high-dose vitamin B1 reduces proliferation in Panc-1 PC cell lines. However, whether or not the consumption of vitamin E, vitamin B12, nicotinic acid, riboflavin, and thiamine can reduce the incidence of PC need more evidence from prospective studies.

Several studies have investigated the mechanism of how vitamins might inhibit PC. Vitamin D can up-regulate p21 and p27 during growth inhibition of PC cell lines.^[5]^ Vitamins A, C, and E can increase the activity of superoxide dismutase to decrease the incidence of PC in hamsters.^[6]^ Vitamin E can induce cell cycle arrest at the G1 phase, induce apoptosis in human PC cells,^[7]^ induce Bax expression, and activate EGR-1 in PC cells.^[11]^ Another study found that vitamin E can inhibit the growth of human PC cells by suppressing NF-κB-mediated inflammatory pathways.^[8]^ Vitamin E can also induce apoptosis in PC cells by suppressing signaling pathways such as the PI3K/AKT and ERK/MAPK pathways via down-regulation of Her2/ErbB2,^[9]^ and inhibit the proliferation of PC cells dependent on p27 (Kip1) induction.^[59]^ Vitamin K can inhibit PC cell survival via a caspase-dependent pathway.^[60]^ Thiamine can increase caspase-3 activity and reduce proliferation in PC cell lines.^[10]^ Nevertheless, the mechanisms of vitamins reducing the risk of PC needs further investigation.

## 5 Conclusion

In conclusion, this meta-analysis suggested that vitamin intake can moderately reduce the risk of PC, particularly the consumption of vitamin D and vitamin B12.

## Author contributions

**Data curation:** S. Lu, X. Sun.

**Funding acquisition:** X. Wang.

**Investigation:** S. Liu, X. Wang, Y. Liu.

**Methodology:** X. Sun.

**Software:** S. Lu, X. Sun.

**Supervision:** Y. Liu.

**Writing – original draft:** S. Liu.

**Writing – review & editing:** S. Liu.
